# Interventions to reduce self-harm on in-patient wards: systematic review

**DOI:** 10.1192/bjo.2021.41

**Published:** 2021-04-16

**Authors:** Rasanat Fatima Nawaz, Gurpreet Reen, Natasha Bloodworth, Daniel Maughan, Charles Vincent

**Affiliations:** Department of Experimental Psychology, University of Oxford, UK; Patient Safety Collaborative, Oxford Academic Health Science Network, UK; and Department of Psychiatry, University of Cambridge, UK; Department of Experimental Psychology, University of Oxford, UK; and Oxford Healthcare Improvement Centre, Oxford Health NHS Foundation Trust, UK; Department of Experimental Psychology, University of Oxford, UK; and Oxford Healthcare Improvement Centre, Oxford Health NHS Foundation Trust, UK; Oxford Healthcare Improvement Centre, Oxford Health NHS Foundation Trust, UK; Department of Experimental Psychology, University of Oxford, UK; and Oxford Healthcare Improvement Centre, Oxford Health NHS Foundation Trust, UK

**Keywords:** Self-harm, in-patient treatment, psychiatric nursing, primary care, education and training

## Abstract

**Background:**

Incidents of self-harm are common on psychiatric wards. There are a wide variety of therapeutic, social and environmental interventions that have shown some promise in reducing self-harm in in-patient settings, but there is no consensus on the most appropriate means of reducing and managing self-harm during in-patient admissions.

**Aims:**

To review interventions used to reduce self-harm and suicide attempts on adolescent and adult psychiatric in-patient wards.

**Method:**

A systematic literature search was conducted between 14 March 2019 and 25 January 2021 using PsycINFO and Medline (PROSPERO ID: CRD42019129046). A total of 23 papers were identified for full review.

**Results:**

Interventions fell into two categories, therapeutic interventions given to individual patients and organisational interventions aimed at improving patient–staff communication and the overall ward milieu. Dialectical behaviour therapy was the most frequently implemented and effective therapeutic intervention, with seven of eight studies showing some benefit. Three of the six ward-based interventions reduced self-harm. Two studies that used a combined therapeutic and ward-based approach significantly reduced self-harm on the wards. The quality of the studies was highly variable, and some interventions were poorly described. There was no indication of harmful impact of any of the approaches reported in this review.

**Conclusions:**

A number of approaches show some promise in reducing self-harm, but the evidence is not strong enough to recommend any particular approach. Current evidence remains weak overall but provides a foundation for a more robust programme of research aimed at providing a more substantial evidence base for this neglected problem on wards.

Self-harm describes the action of intentionally injuring or poisoning oneself, regardless of motivation or suicidal intent.^1^ Types of self-harm can be divided into two groups: self-injury (including such acts as self-cutting, ligature-tying and self-battery) and self-poisoning (by taking an overdose of legal prescription or over-the-counter medications, for example, analgesics such as paracetamol). Risk factors for self-harm with both non-suicidal and suicidal intent include age, gender, mental health diagnosis, coping strategies, previous self-harm, acute stress response, relationship with family and friends, as well as social deprivation.^2–7^ The risk of attempted and completed suicide is significantly higher in those who have engaged in self-harm,^8–10^ with one study estimating that 50% of young people that had completed suicide would have previously self-harmed.^11^ A history of self-harm has been demonstrated to be one of the primary indicators of completed suicide.^12^

## Self-harm on wards

In-patient services are highly specialised, reserved for the treatment of patients with the most severe mental health disorders.^13^ Unsurprisingly, incidents of self-harm are relatively commonplace on a psychiatric in-patient ward.^14^ Around 10–20% of adolescent in-patients will self-harm at least once during their stay, and a proportion of these will self-harm repeatedly as many as 130 times.^15–18^ In addition, risk of suicide is increased soon after admission and immediately after discharge.^19^ The prevention of self-harm and suicide is one of principal preoccupations and primary roles of ward staff in a mental health setting.^20^

The in-patient environment has additional unique factors that can contribute to self-harming. Patients may be distressed by interaction with other in-patients, by the rules and routines of life on the ward, the amount of leave granted or by the restrictions of involuntary admission.^3,21,22^ Feeling lonely, being isolated from others and a lack of stimulation can all contribute to self-harming behaviours on psychiatric wards.^23,24^ Patients may use self-harming behaviour as a way of seeking help when they do not feel supported by nursing staff on wards.^25,26^

Patients at risk of self-harm are almost always placed under constant observation. A nurse is required to observe the patient at all times, as they are thought to be at risk of suicide, self-harm or violent behaviour,^27^ although wide variations in actual practice of constant observation exist.^28^ The key purpose of increased observation is to provide a period of safety for people during temporary periods of distress when they are at risk of harm to themselves and/or others. Observation is, however, potentially distressing and personally invasive for patients, burdensome and time-consuming for staff and can still result in death by suicide for the patient.^29,30^

Restraint and seclusion are commonly used to manage self-harm behaviours, and other behaviours that pose a significant threat to other patients or staff members.^31,32^ However, such restrictive practices may lead to physical injury to both staff and patients,^33^ as well as the potential of significant negative psychological effects on staff and patients.^34^ Consequently, there have been drives to reduce the use of restrictive practice except in the most serious of incidents and to explore more humane and ethical ways of reducing self-harm.

## Reducing self-harm on psychiatric wards

There is a major question in current clinical practice as to whether admission to a psychiatric ward should be used to manage self-harm or suicide risk. A large proportion of patients presenting with these problems have a diagnosis of emotionally unstable personality disorder (EUPD).

The National Institute for Health and Care Excellence guidance for managing EUPD,^35^ states that clinicians should ‘explore other options before considering admission to a crisis unit or in-patient admission’. Further, longitudinal data-sets suggest that admission to hospital does not reduce the risk of suicide, and multiple admissions to manage suicide risk is associated with an increased risk.^36^ However, where the self-harm or suicide risk is associated with a mental illness that can be treated using medications, such as bipolar disorder, depression or schizophrenia, a short-term admission for medical treatment is clearly justifiable as the risk of self-harm reduces substantially following treatment.^36^ The management of self-harm and suicide risk on psychiatric wards will therefore need to be addressed on wards, whether or not these risks were the major reason for admission. Interventions to address these risks therefore remain pertinent.

Therapeutic approaches such as dialectical behaviour therapy (DBT) and cognitive–behavioural therapy (CBT), and emotion-regulation training may all be used to treat adolescents who self-harm. Both DBT and CBT have been adapted to in-patient settings and have been shown to reduce self-harming behaviours in adolescents.^37–41^ Treatment in out-patient settings is usually intensive and relatively prolonged, so the impact of treatment during a short in-patient admission is likely to be modest. Social and environmental factors also play a role in increasing the likelihood of self-harm. These factors include relationships between patients and staff, relationships with other patients, the physical environment and the organisation of care.^42,43^ These wider causes and influences can also be addressed in social and organisational interventions such as adjusting staff rotas to cover high-risk periods and providing additional interests and social activities.^29^ Such interventions, in the context of an in-patient environment, have the advantages of a relatively immediate impact, providing a more positive atmosphere for both patients and staff and potentially reducing the need for observation, restraint and seclusion.

## Aims

In summary, there are a wider variety of therapeutic, social and environmental interventions that have shown some promise in reducing self-harm on in-patient wards. However, the studies vary considerably in methodology, the evidence is scattered across many different journals and disciplines and there is no consensus on the most appropriate means of reducing and managing self-harm during an in-patient admission.

The present systematic review considers interventions that may be used to reduce the incidence and severity of self-harm and suicide attempts in adolescent and adult psychiatric in-patient settings. Our aim was to assess the efficacy of interventions and provide guidance to researchers on which interventions show promise and deserve further study, and provide guidance to clinical teams on which interventions are most effective in reducing self-harm on in-patient wards.

## Method

The preferred reporting items for systematic reviews and meta-analyses (PRISMA) recommendations were used as guidelines to report this review.^44^ A protocol for the review was registered with PROSPERO (ID: CRD42019129046).

### Systematic literature search

The systematic literature search was conducted between 14 March 2019 and 25 January 2021 using PsycINFO and Medline. Additional relevant articles were also identified by screening references and reading titles and abstracts of papers in relevant journals. Search terms were developed by authors and can be accessed in the Supplementary Table 1 available at https://doi.org/10.1192/bjo.2021.41.

### Eligibility criteria

The following criteria had to be met for studies to be included in the review: peer-reviewed, any date, in English, any country, any study design, must be evaluated, and have on an impact in-patient self-harm behaviour on a ward. Self-harm was defined as any act of self-poisoning or self-injury carried out by a person, including self-harm with or without suicidal intent. Interventions with any aim were included if impact on self-harm was reported as an outcome measure. Only interventions conducted primarily in in-patient settings were included. All in-patient settings were included for example forensic, psychiatric intensive care units (PICUs) and adolescent wards. No restrictions were placed on patient's age, ethnicity, demographics or the presence and nature of any psychiatric conditions.

Exclusions included studies that examined self-harm reduction interventions in the emergency department as well as other general hospital settings. Studies were also excluded if they were qualitative, commentaries, reviews or were about people with intellectual difficulties as the aetiology of self-harm is different in this group.^45^ Studies conducted only in community psychiatric settings were excluded, unless parts of the intervention were also implemented in an in-patient psychiatric ward.

### Screening and data extraction

Author-developed data extraction forms were used to extract relevant information and data from the full-text articles. The data extraction forms were based on the Template for Intervention Description and Replication.^46^ The forms included, where available: the aim of the intervention, design and development, type of in-patient setting, study design, baseline data, control group, duration and frequency, outcome measures, results and limitations identified by authors, description of the intervention, year of implementation, type of ward, description of ward, country, patient and staff demographics and characteristics.

Initial extraction was carried out for five papers by one reviewer (R.F.N.) and checked by a second reviewer (G.R.) who has extensive experience in conducting systematic reviews. Any discrepancies between the authors were resolved through discussion. Following this, the remaining papers were extracted by R.F.N.

### Quality assessment

Quality ratings were completed for all 23 studies, using the Effective Public Health Practice Project (EPHPP) quality assessment tool. Quality rating was conducted by two authors (R.F.N. and G.R.) and any discrepancies in ratings were resolved through discussion. This tool is suitable to assess quality across different study designs conducted in healthcare settings. This tool has been used in multiple healthcare-related systematic reviews.^47,48^ The final quality rating of each study was derived from the ratings on the following six measures: selection bias, study design, confounders, masking, data collection methods and withdrawals or drop-out. No studies were excluded from the review based on quality ratings.

### Data synthesis

As a result of high heterogeneity of study designs, interventions and outcome measures, it was not appropriate to conduct a meta-analysis. Studies were grouped into either therapeutic, ward based or mixed. Therapeutic studies were interventions aimed at improving the well-being of patients through therapy. Ward-based studies were interventions targeting the ward environment and ward milieu, to reduce self-harm. Studies that used both therapeutic and ward-based interventions were grouped under mixed intervention.

## Results

**Fig. 1:**
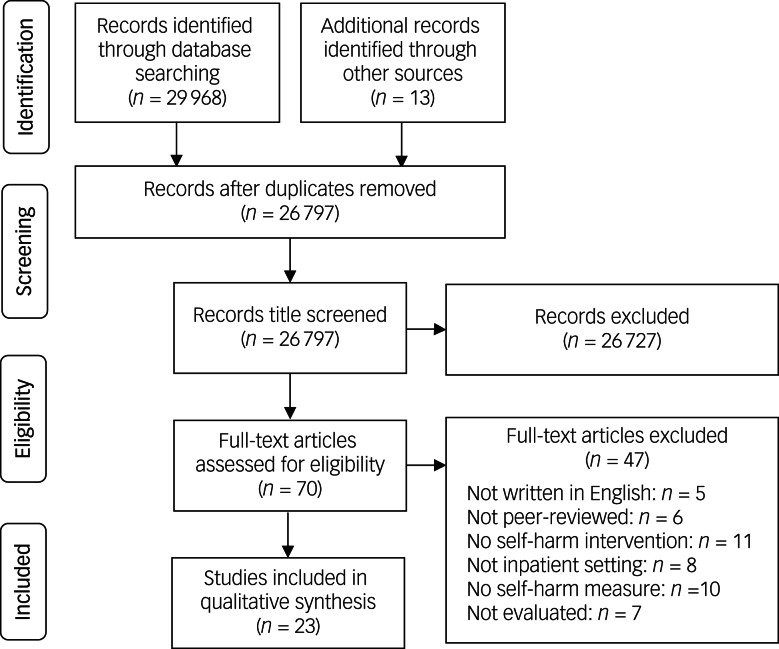
PRISMA flow chart for selection process of studies in the systematic review.

The initial search generated 29 968 articles across PsycINFO and Medline, additional screening of reference lists identified 13 more papers. Using reference managing software, duplicates were removed, and article titles were screened (*n* = 26 797). A total of 70 papers were eligible for full-text review. For each stage of the screening process, the number of articles is presented in Fig. 1. A total of 23 full-text papers were identified and included in the final review. See Supplementary Table 2 for a breakdown of the characteristics of all 23 studies.

### Study characteristics

#### Study setting

A total of 23 studies evaluated an intervention to reduce self-harming behaviour on in-patient psychiatric wards and were included in the present review. The majority of the studies were conducted in the USA (*n* = 11),^49–59^ followed by the UK (*n* = 6),^43,60–64^ Germany (*n* = 2),^65,66^ Ireland (*n* = 2),^67,68^ Italy (*n* = 1),^69^ and Pakistan (*n* = 1).^70^

Interventions were conducted on both adult wards^49,50,54,56,58–70^ and adolescent in-patient wards.^43,51–53,55,57^ Many wards did not have any gender restrictions (*n* = 10),^49,50,53–56,58,60,69,70^ but a few studies conducted interventions on female-only (*n* = 3)^62,65,66^ or male-only psychiatric ward (*n* = 1),^63^ or did not report the gender of the ward (*n* = 7).^52,57,59,61,64,67,68^ In total, 62 in-patient psychiatric wards were included in the present review. Many of these were acute psychiatric wards (*n* = 41),^49,54,56,58–63,65–70^ followed by forensic (*n* = 6),^64^ child and adolescent wards (*n* = 7),^43,51–53,55,57^ PICU (*n* = 4)^60^ and triage/assessment units (*n* = 3).^60^ One study did not report the number or type of wards.^50^

#### Study aims and design

The study designs varied with the majority using a pre and post design (*n* = 14),^49,51–53,55,57–59,61–63,65,66,69^ followed by randomised controlled trials (*n* = 6),^50,54,56,60,68,70^ non-randomised controlled trials (*n* = 2)^64,67^ and interrupted time series (*n* = 1).^43^ The majority of the studies had control groups except five.^57,59,61–63^ All interventions aimed to reduce the frequency of self-harm, although many also monitored depression, anxiety, suicidal ideation and other indices.

#### Quality ratings

All 23 studies were rated for quality using the EPHPP tool. One intervention had a strong quality rating,^60^ and five studies had a moderate quality rating.^49,50,54,65,70^ The remaining 17 studies were rated as weak because of selection bias and masking.^43,51–53,55–59,61–64,66–69^ See Supplementary Table 3 for a full breakdown of quality ratings.

### Nature of interventions

The majority of the studies were therapeutic interventions for patients (*n* = 15).^49–51,53–58,65–70^ A smaller selection of studies implemented interventions focusing on changes to the ward environment (*n* = 6).^52,59–61,63,64^ Two studies used a combination of therapeutic and ward-based techniques to develop and implement an intervention to reduce self-harm.^43,62^ See Table 1 for descriptions of the interventions.

**Table 1:** Descriptions of therapeutic interventions, ward-based interventions and mixed interventions

Intervention type	Intervention details
**Therapeutic interventions**	
Dialectical behaviour therapy (DBT) (*n* = 8)	DBT for female in-patients with emotionally unstable personality disorder (EUPD), 3 months treatment^65,66^
	DBT for 6 weeks^67^
	DBT for adolescent psychiatric in-patient unit – 2 weeks, 10 sessions^53^
	DBT with three different intensity levels in a psychiatric in-patient setting, length not stated^55^
	DBT and cognitive–behavioural therapy (CBT) for in-patients with a personality disorder, 10 sessions, 45 min daily^56^
	Modified psychodynamic treatment programme to emphasise DBT use for patients with borderline personality disorder^58^
	DBT implementation for adolescents, 9 sessions per week^51^
Problem-solving therapy (*n* = 2)	Culturally adapted problem-solving therapy, 6 sessions, 3 months in Pakistan^70^
	Group problem-solving skills training for self-harm, six 2 h sessions^68^
Skills to enhance positivity (STEPs) and and Systems Training for Emotional Predictability and Problem Solving (STEPPS) therapy (*n* = 2)	A two-part positive affect programme (STEPPS) designed to decrease recurrent suicidal behaviour, 20-week programme for patients with EUPD^69^
	Modified skills to enhance positivity (STEPs) therapy, participants picked own topics to cover, with technology-assisted reminders on ward and 4 weeks of daily text messages post-discharge^57^
Unified protocol (*n* = 1)	Unified protocol is a cognitive–behavioural intervention addressing psychological disorders with aversive reactions to frequent negative emotion. Unified protocol (5 sessions) was modified to address suicidal thoughts and behaviours in an in-patient setting^49^
Phone-based positive psychology (*n* = 1)	Phone-based positive psychology, weekly one-on-one telephone sessions over 6 weeks^50^
Post-admission cognitive therapy (PACT) (*n* = 1)	PACT is an in-patient CBT programme based on the cognitive model of depression and suicide. Present study used this on military personnel with post-traumatic stress disorder, 60–90 min CBT sessions over 3 days^54^
**Ward-based interventions**	
Patient–staff communication, including Safewards: (*n* = 3) Staff training (*n* = 3)	Safewards, a package of 10 interventions focusing on patient-centred care and behaviour standards for staff and patients in order to reduce containment rates^60^
	Safewards, exploring the effect of this 10-part intervention on 6 forensic wards, 10-week intervention^64^
	Reduce and replace constant observations and promote professional culture^63^
	Employing clinical experts. Nurses worked with ward staff 3 days a week to move towards low-conflict low-containment therapy-based nursing^61^
	Alternative to constant observation using 17-point behavioural checklist^59^
	Collaborative problem-solving, using retrospective staff surveys and hospital medical records to compare behavioural outcomes and staff perceptions during pre- and post-training phases of a 5-year study^52^
**Mixed interventions**	
Zonal nursing and therapeutic days (*n* = 1)	Using zonal nursing where staff are allocated to specified zoned areas in the ward rather being assigned to an individual patient. Patients can move between areas and be monitored discretely. Patients were also involved in co-designing therapeutic days, including recreational, therapeutic and physical activities^62^
Additional nurse and activities (*n* = 1)	Regular twilight shift (15.00–23.00 h) and structured evening activities programme for in-patient adolescents^29^

#### Length of interventions

The length of the interventions varied considerably across the 23 studies. The length for the 14 therapeutic interventions varied: 3 days,^54^ 4 days,^49^ 10 days,^56^ then 1 month,^50,57,67,68^ 2 months,^64^ 3 months,^58,65,66,70^ 12 months^55^ and 18 months^63^. The six ward-based interventions generally lasted longer with 2 weeks,^53^ 4 months,^59,69^ 6 months,^60^ 12 months^61^ and 5 years.^52^ The interventions using a mixed approach lasted 12 months^62^ and 18 months.^43^

#### Development of interventions

The majority of interventions were developed using staff and patient interviews^43,59,61,62^ or the interventions were existing therapies adapted specifically for patients with EUPD,^56,58,66,69^ patients with major depression^50^ or to suit the in-patient environment.^51,53–55,65–67^

### Patient demographics

A total of 2402 patients were included in the studies under review. Five studies did not report participant sample size, but instead reported number of wards only.^59–61,63,64^ Over half (73%) of the participants were female patients (*n* = 1184) and 27% were male patients (*n* = 430). Eight studies did not report the gender of patients.^52,57,59,61,63,64,67,68^ Participants were aged between 12 and 18 years on adolescent wards and 16 and 70 years on adult wards, with seven studies not reporting the age of patients.^56,59,61–64,67^

Some studies reported patient diagnoses and employed interventions that were adapted to suit patients with specific illnesses. These included interventions aimed at patients with borderline personality disorder^56,65,66,69^ and major depression,^50^ interventions aimed at patients with a history of self-harm and suicide ideation,^53,54,67,68,70^ and two studies were open to patients with any diagnoses.^49,55^ Eight studies did not report patient diagnoses.^52,57–64^

### Measures (baseline and follow-up)

Baseline measures varied across the studies, with four studies not reporting a baseline measure.^51,53,55,63^ All but seven studies had a follow-up period.^43,51,55,56,61,63,64^ Eight studies had multiple follow-up periods.^49,50,54,57,62,68–70^ Follow-up times varied from 1 month (*n* = 5),^49,54,57,65,66^ 6 weeks (*n* = 2),^50,68^ 2 months (*n* = 1),^54,60^ 3 months (*n* = 6),^50,54,57,62,67,70^ 6 months (*n* = 6),^49,57,59,68–70^ 12 months (*n* = 4),^53,62,68,69^ 14 months (*n* = 1)^58^ to 5 years being the longest.^52^

### Outcome measures

All studies had a self-harm outcome measure and reported a variety of other measures relevant to patients and the in-patient environment. There were also differences in the measures used when reporting therapeutic interventions versus reporting ward-based interventions.

#### Self-harm outcome measures

The most common self-harm outcome measures for therapeutic interventions included self-harm reports, these were collected through hospital/ward incident reporting systems. Some studies also looked at suicide ideation by using self-injury questionnaires and standardised scales. However, the scales used varied greatly and some were self-reports such as the Suicide Ideation Questionnaire, Beck Scale for Suicide, Self-Injurious Thoughts and Behaviours Interview–Self-Report, Columbia Suicide Severity Rating Scale and the Lifetime Parasuicide Count. Many studies grouped non-suicidal self-harm and self-harm with suicidal intent and did not justify why a certain measure/tool was used over another.

Studies implementing ward-based interventions primarily collected self-harm data and number of suicide attempts through hospital/ward incident reporting systems. A few studies also developed a one-page checklist for clinical staff to record a range of conflict incidents, including self-harm.

#### Other outcome measures

Additional outcome measures for therapeutic interventions included adherence to intervention, changes in medication and changes in mood. Depression and anxiety were measured using different inventories across the studies. These included: the Beck Depression Inventory, Hamilton Rating Scale for Depression, Beck Anxiety Inventory, Hamilton Rating Scale for Anxiety and State–Trait–Anxiety Inventory.

For ward-based interventions, additional measures included number of hours patients spent under constant observation, staff sickness, staff budgets, other harmful patient incidents (e.g. violence and aggression), and incidents of restrictive practice (e.g. restraint). These additional measures were not present in the therapeutic interventions.

### Impact on self-harm

In total, 15 of the 23 interventions^43,51–53,55–58,61,63,65,66,69–71^ showed a statistically significant reduction in self-harm. Six studies^50,54,60,64,67,68^ did not show a significant reduction in self-harm and two studies^49,59^ did not report outcome data despite reporting they collected self-harm incident data.

For the 15 therapeutic interventions, 10 studies^51,53,55–58,65,66,69,70^ showed a significant reduction in self-harm, suicidal ideation and suicide. Five studies^49,50,54,67,68^ failed to show a significant effect. No studies showed an increase in self-harm incidents following therapeutic interventions.

For the six ward-based interventions, three studies^52,61,63^ showed a significant reduction in self-harm and three did not.^59,60,64^ Both mixed interventions^43,62^ using a combined therapeutic and ward-based approach significantly reduced self-harm. Specific therapeutic and organisational approaches are discussed in more detail below.

### Therapeutic interventions

#### DBT

DBT was adapted to in-patient settings in different ways across eight studies and there were numerous differences in implementation and measurement of outcomes. Seven^51,53,55,56,58,65,66^ out of the eight studies significantly reduced self-harm.

Five studies^56,58,65–67^ used adapted in-patient DBT for patients with EUPD, who had a history of self-harm. One randomised controlled trial^56^ used 45 min CBT and DBT group sessions for 31 patients, administered over 10 days by experienced nurses. The control group was an existing wellness group on the ward, however, there was no further follow-up. The outcomes included a significant reduction in self-harm, and parasuicide. Another two studies administered standard DBT over 3 months by trained staff, using a pre–post design in 24 patients^66^ and 60 patients.^65^ Both these studies showed a significant reduction in self-harm acts. One study^67^ implemented DBT over 6 weeks and showed a reduction in self-harm by comparing group means but it was not clear whether this was statistically significant. Another study^58^ adapted a psychodynamic approach to include DBT in the personality disorders treatment programme for 3 months and found a significant reduction in self-harm when compared with the control group.

Three studies^51,53,55^ adapted DBT for adolescent in-patients and showed a statistically significant reduction in self-harm incidents. In one study,^53^ DBT was adapted for 2 weeks and provided to 32 participants. This study found a significant reduction in self-harm on the wards compared with the baseline, improvements continued at a 12-month follow-up but these were not statistically significant. The second study^55^ adapted DBT for adolescents in a long-term in-patient facility, showing a significant reduction in non-suicidal self-harm when compared with historical controls. The sample size was larger with 210 participants. The intervention lasted 12 months and included all components of the DBT model. The third study^51^ evaluated DBT adapted for adolescents versus treatment as usual for adolescents on an acute-care psychiatric in-patient unit.

#### Skills to enhance positivity (STEPs) therapy and Systems Training for Emotional Predictability and Problem Solving (STEPPS) therapy

Two studies used Skills to Enhance Positivity (STEPs) and Systems Training for Emotional Predictability and Problem Solving (STEPPS) therapy, which led to statistically significant reductions in the number of hospital admissions for self-harm acts^69^ and suicidal ideation.^57^ STEPPs was originally intended for out-patients with EUPD. One study^69^ tested the standard STEPPs manual in the in-patient environment for 32 patients with EUPD over 20 weeks. The second study^57^ modified STEPs for 20 adolescents over 1 month and allowed participants to select topics, such as meditation and gratitude, to cover within each session, encouraging buy-in. Both studies used a pre and post design, only one of the studies^69^ had a control.

#### Problem-solving therapy

Two studies^68,70^ implemented problem-solving therapy using a randomised controlled trial design. First, a culturally adapted problem-solving therapy for 221 patients, lasting 3 months, significantly reduced suicide ideation.^70^ Second, a group problem-solving intervention for 433 patients, lasting 6 weeks, had no impact on rates of self-harm at 6-week follow-up, 6-month follow-up and 12-month follow-up.^68^ It is unclear why these differences in outcomes occurred, this could be because of the length of the interventions or even that the culturally adapted version^70^ was being implemented in a hospital where little support was in place for self-harm.

#### Other therapeutic interventions

The remaining therapeutic interventions did not have a significant impact on reducing self-harm incidents. Post-admission cognitive therapy showed no significant differences in reducing suicide risk when compared with the control.^54^ An adapted unified protocol intervention reported a difference in means for suicide ideation but the sample was too small to conduct further analyses.^49^ Phone-based positive psychology showed a significant difference in favour of the control group, in suicidality, depression and hopelessness at 6 weeks and 12 weeks.^50^

### Interventions to provide a safer ward environment

#### Patient-staff communication: ‘Safewards’ ten-component intervention

The Safewards intervention^60^ consisted of ten components that, broadly speaking, aim to improve relationships between patients and staff, foster a safe atmosphere on the ward and respond rapidly to signs of agitation or distress. Staff are, for instance, encouraged to talk to patients informally and patient groups and activities provide interest and social relationships. A concerted attempt is also made to highlight positive achievements, positive messages about the ward and those patients who have successfully returned to the community.

The Safewards intervention was conducted on multiple wards using a randomised controlled trial study design. Two replications of this intervention showed mixed results. One study,^63^ with a pre and post design, showed a significant reduction in suicide and self-harm. The other study^64^ was a non-randomised controlled trial and did not show significant reduction in self-harm incidents.

#### Staff training

Two out of the three studies focusing on staff training significantly reduced self-harm incidents. One study^61^ employed two additional nurses to work on two acute wards for 1 year, assisting with the implementation of changes according to a working model of conflict and containment. A retrospective study^52^ found collaborative problem-solving training for nurses led to a significant decrease in self-harm incidents. The data spanned 5 years and included staff surveys and hospital medical records over a 5-year span. Collaborative problem-solving taught staff to diffuse tenuous situations for example by distracting or engaging the patient in respectful conversation. The final study^59^ introduced an alternative checklist to constant observation, the researchers collected self-harm data but did not report the findings.

### Mixed intervention

One intervention^62^ combined both a therapeutic approach and ward-based changes. This was done through changing how nurses were placed on the ward, placing boundaries and time restrictions for certain ward areas, and introducing recreational, physical and therapeutic activities for in-patients. This intervention showed a significant reduction in self-harm, and the effects were sustained over 12 months.

The second mixed intervention^43^ involved adding a regular twilight shift (3–11 pm) for nursing staff and introducing a structured evening activity programme on the ward. A tailored intervention targeting the psychiatric ward environment helped to reduce the proportion of adolescents self-harming on the ward.

## Discussion

The management and reduction of self-harm is a priority for all psychiatric wards.^20^ In spite of this, we identified only 23 studies evaluating self-harm reduction on wards of which only 15 reduced self-harm.

### Efficacy of specific therapeutic and organisational interventions

DBT was the most frequently implemented and effective intervention, with seven of eight studies showing some benefit. Sample sizes varied considerably, from 24 patients in the smallest studies to 425 in the largest. Length of treatment also varied; two studies^65,66^ lasted 3 months and showed significant reduction in self-harm, but modified DBT programmes also reduced self-harm on adolescent wards.^51,55^ A systematic review performed in 2016^72^ on interventions for self-harm in the community found that CBT reduced overall numbers of self-harm, whereas DBT did not. However, DBT was found to reduce frequency of self-harming within individuals. In this 2021 review, only two studies were identified as using some form of CBT , and on its own CBT did not seem to have an impact on self-harm on the ward. This suggests that DBT may be a more promising therapy for patients on psychiatric wards, whereas CBT should be reserved for patients in the community. However, additional controlled trials are needed to examine this further.

Three out of the six ward-based studies used ‘Safewards’, a combination of ten practices that were aimed at improving communication between staff and patients on an in-patient psychiatric ward, but only one demonstrated a significant reduction in self-harm.^63^ The different results for the same interventions were likely because of the difficulties in implementing ten practices in quick succession without adapting to the needs of the local environment rather than the nature of the intervention itself. This was noted by clinicians on psychiatric wards where the intervention was not successful.^64^

Of the remaining three ward-based interventions,^52,59,61^ two interventions^52,61^ managed to reduce self-harm by improving the ward milieu by training staff on how to manage difficult patient behaviours. Interventions published since this review was conducted have further shown reductions in self-harming behaviours following tailored improvements to the ward environment.^43^ There are a number of ways that ward-based interventions could have an impact on patient self-harm. These include, increasing the presence of staff available for patients to seek help and feel supported,^22,73^ providing positive and safe ways to distract patients and help them to bond with their peers to replace the positive functions associated with self-harm.^74–76^ In addition, ward-based activities and interventions may help to reduce the sense of isolation, restriction and loneliness that patients often feel on psychiatric wards that may trigger self-harming behaviours.^77^ This is a promising finding as ward-based interventions are often quicker to implement, can be tailored to the ward environment, and are likely to have a positive impact on other aspects of the ward.

The two studies^43,62^ in this review that used a combined therapeutic and ward-based approach significantly reduced self-harm on a ward, and these effects were sustained over time. In one^62^ of these studies, all patients were provided with individual therapy and all staff were given specific training to manage self-harm and other difficult behaviours. The second intervention also found that introducing activities and a regular twilight shift in the evening, when self-harming behaviours were increased, successfully reduced self-harm on the ward.^43^

### Limitations

The quality of the studies was highly variable, with only one study having a strong design. Most studies used simple pre–post designs with small sample sizes, and several were without controls of any kind. Many interventions were complex, not well defined and poorly described. A wide variety of therapeutic and organisational interventions were employed; none of the single interventions showed a consistent outcome. The data in this review does not distinguish between number of self-harming incidents overall and the number of individuals self-harming because of the lack of information provided by the studies. Therapeutic interventions for individual patients were more common than organisational interventions aimed at improving the ward milieu and proactive management of self-harm.

Some studies took place in private hospitals/wards where there are very different demands and constraints on both patients and staff compared with the public sector. These differences may influence the cohort being studied, the length of admission, the number of staff on the ward, the ward culture and other activities available on the ward. There is likely to be further variation between different organisations and particularly between countries. However, there are not sufficient studies available across different countries to draw any conclusions about what approaches might suit particular cultures and contexts. This issue of widely differing ward contexts serves to undermine the generalisability of results from any particular setting.

The short timescales of some therapeutic studies included in this review are particularly problematic, given the intractable nature of self-harm. Self-harming behaviours are complex in how they are caused and maintained.^25^ Short time frame interventions on wards are unlikely to be of lasting benefit unless combined with longer-term and continuing therapy in community settings.

### Clinical implications

The findings from this review suggest that a variety of approaches show some promise in reducing self-harm, but the evidence is not strong enough to recommend any particular approach. However, several approaches appear to provide benefit and there was no indication of a harmful impact of any of the approaches reported in this review. The mixed interventions^43,62^ addressing individual therapy alongside ward management and staff training were effective at reducing self-harm in the short and longer term.

Many factors influence the likelihood of self-harm, with different influences on different people. Some patients for instance may be primarily responding to longstanding personal distress and difficulties, while others may be more influenced by current social relationships and the immediate ward environment. This suggests that clinical teams should consider individual therapeutic approaches combined with social and organisational interventions. This is supported by a recent study that found that ward-based interventions could reduce the number of people that self-harm, but did not have an impact on the most vulnerable patients with repeated self-harm incidents who may have benefited from an additional therapeutic intervention.^43^ At the very least, wards should prioritise proactive management of self-harm and target the therapeutic support to the most vulnerable patients given the potential benefits to the patient, and others on the ward, and the risk of suicide associated if this is not addressed.

A critical question that needs addressing, is whether patients should be admitted with a primary aim of reducing self-harm or suicide risk when there is no other clear treatment goal. Current guidance for managing self-harm or suicide risk suggests admissions are ideally avoided^35^ given the potential adverse consequences of admission^36^ and the costs of in-patient care. Quality standards for in-patient care^78,79^ do not recommend any particular approach to managing self-harm. It is evident from the findings of this review that this may be a result of the paucity of evidence available.

The evidence presented in this systematic review is not sufficient to recommend that admission should be used as a first-line treatment to manage self-harm in isolation. However, self-harm and suicide risk remains a core feature of many psychiatric patients who require admission for other reasons. The studies presented here, although they have many limitations, do offer potential directions and guidance for wards in showing how they might reduce risk of self-harm.

### Future research

The interventions used to reduce self-harm are remarkably diverse, with few studies appearing to build on previous experience and earlier findings. Future studies aimed at reducing self-harm need to describe interventions in more detail and to have a clearly defined rationale for reducing self-harm. Studies should include control groups, larger sample sizes and a range of standardised outcome measures to best understand the impact.

Mixed interventions, which combine individual therapeutic work with wider ward interventions, may hold promise. The idea being that multiple mechanisms are involved in self-harm and therefore different strategies are likely to be complementary. Such approaches could be effectively studied with cluster-randomised trials at ward level or potentially in a stepped-wedge design as the intervention is implemented across different wards and organisations.

There remains a question about whether DBT can be more effective if initiated in an in-patient setting and then continued in a community setting. Short-term DBT customised to an in-patient setting could be effective but the impact can be short lived if not followed up with longer-term therapeutic engagement in the community. Trials are needed to gain a better understanding of the role of DBT in reducing self-harm on wards and to compare different patterns and timing of treatment. There is also a clear need for more interventions looking at the effectiveness of CBT in managing self-harm on the ward given that this was found to be the most effective treatment for stopping self-harm in the community.^72^

In conclusion, self-harm is traumatic for individuals and families and has an associated risk of suicide in the longer term. The management of self-harm using observations and restraint is seen as invasive by patients and consumes inordinate amounts of clinical time.^80,81^ There is an urgent need to find effective, evidence-based ways of managing self-harm in a ward environment that provides both immediate benefit to patients and a foundation for longer-term therapeutic impact. Current evidence reviewed here remains weak overall but most interventions in this review show benefit; however, more robust programmes of research are needed to provide a more substantial evidence base for this neglected problem on psychiatric in-patient wards.

## Data Availability

Data availability is not applicable to this article as no new data were created or analysed in this study.

## Supplementary material

For supplementary material accompanying this paper visit http://dx.doi.org/10.1192/bjo.2021.41. click here to view supplementary material
