# Supplementary material for: Interventions to reduce self-harm on in-patient wards: systematic review
Source: BJPsych Open. 2021 Apr 16;7(3):e80. doi: 10.1192/bjo.2021.41 (PMC8086389; doi:10.1192/bjo.2021.41)
Supplement: Supplementary file 1 [file S2056472421000417sup001.docx]

| Table 3: Search terms to shortlist studies for review | |
| --- | --- |
| Database | Search Terms |
| PsycINFO | Self-destruct$ OR self-harm$ OR self-injur$ OR self-mutilate$ OR self-poison$ OR self-inflict$ OR self-mutilat$ OR conflict OR deliberate self-harm$ OR DSH OR parasuicide$ OR para suicide$ OR suicid$ attempt$ OR suicid$ episode$ OR suicid$ frequen$ OR suicid$ recur$ OR suicid$ repeat$ OR suicid$ repetition OR overdos$ OR over dos$ OR auto mutilat$ OR automutilat$ OR cutt$ OR head bang$ OR self-cut$  AND Inpatient$ OR in-patient$ OR ward$ OR department OR service user OR service-user OR hospital |
| Medline |  |
|  |  |
|  |  |
|  |  |
|  |  |
|  |  |

Table 4: Quality assessment of evaluated interventions (n=21)

| Study (first author, year) | Selection bias | Study design | Confounders | Blinding | Data collection method | Withdrawals and dropout | Overall quality rating |
| --- | --- | --- | --- | --- | --- | --- | --- |
| Alesiani et al 2014 | Weak | Moderate | Weak | Weak | Strong | Weak | Weak |
| Barley et al 1993 | Moderate | Moderate | Weak | Weak | Strong | Weak | Weak |
| Bentley et al 2017 | Moderate | Moderate | Strong | Weak | Strong | Strong | Moderate |
| Bohus et al 2000 | Moderate | Moderate | Weak | Weak | Strong | Moderate | Weak |
| Bohus et al 2004 | Moderate | Moderate | Strong | Weak | Strong | Moderate | Moderate |
| Bowers et al 2006 | Weak | Moderate | Strong | Weak | Strong | Moderate | Weak |
| Bowers et al 2015 | Strong | Strong | Strong | Moderate | Strong | Strong | Strong |
| Carr 2012 | Moderate | Moderate | Weak | Weak | Moderate | Strong | Weak |
| Celano et al 2017 | Weak | Strong | Strong | Moderate | Strong | Strong | Moderate |
| Dodds 2001; Bowels 2003 | Weak | Moderate | Strong | Weak | Moderate | Strong | Weak |
| Ercole-Fricke et al 2016 | Moderate | Moderate | Weak | Weak | Moderate | Not applicable | Weak |
| Gibson et al 2014 | Weak | Moderate | Strong | Weak | Strong | Moderate | Weak |
| Husain et al 2014 | Moderate | Strong | Strong | Weak | Strong | Strong | Moderate |
| Katz et al 2004 | Weak | Moderate | Strong | Weak | Strong | Strong | Weak |
| LaCroix et al 2018 | Moderate | Strong | Weak | Moderate | Strong | Moderate | Moderate |
| McAuliffe et al 2014 | Strong | Strong | Weak | Weak | Strong | Weak | Weak |
| McDonell et al 2010 | Weak | Moderate | Weak | Weak | Strong | Weak | Weak |
| Moran 1978 | Weak | Moderate | Strong | Weak | Weak | Moderate | Weak |
| Price et al 2016 | Weak | Moderate | Strong | Weak | Strong | Weak | Weak |
| Reen et al 2020 | Moderate | Moderate | Weak | Weak | Weak | Weak | Weak |
| Springer et al 1996 | Weak | Strong | Strong | Weak | Strong | Moderate | Weak |
| Tebbett-Mock et al 2020 | Weak | Moderate | Weak | Weak | Weak | Weak | Weak |
| Yen et al 2019 | Weak | Moderate | Weak | Weak | Strong | Strong | Weak |

Overall quality rating; strong = no weak ratings; moderate = one weak rating; weak = two or more weak ratings

Not applicable = due to retrospective nature of study
